# Advancing Use of Nutrition Knowledge to Improve Practice by Policy and Program Communities in India During a Political Transition

**DOI:** 10.1093/cdn/nzab120

**Published:** 2021-09-20

**Authors:** Edward A Frongillo, Jessica L Escobar-Alegria

**Affiliations:** Department of Health Promotion, Education, and Behavior, University of South Carolina, Columbia, SC, USA; Alive & Thrive at FHI Solutions, Washington, DC, USA

**Keywords:** knowledge, India, nutrition policy, nutrition programs, political transition

## Abstract

Models are needed for how to advance use of knowledge by programs and policy officials to make evidence-based decisions about nutrition. How to advance use of nutrition knowledge in India from 2011 to 2015 during a political transition was investigated through studying a knowledge initiative led by the International Food Policy Research Institute. Semistructured interviews were conducted with 37 diverse participants, and 1091 news articles, 318 Twitter and 175 Facebook posts, 12 YouTube events, 65 knowledge products, and 130 engaging events were assessed. Open-axial coding, content and themes analysis, triangulation, and process tracing were used. The knowledge initiative analyzed the landscape, made knowledge available, mobilized it, and engendered its use. After political transition, knowledge was readied for the reassembled nutrition technical community, including timely responses to governmental information needs. Making nutrition knowledge available, mobilizing it, and engendering its use can be advanced through knowledge initiatives in large, complex countries during political transitions.

## Introduction

Malnutrition has large social and economic consequences affecting nearly all countries (1). Malnutrition takes multiple forms, including undernutrition, overweight and obesity, and micronutrient deficiencies. Nutrition is affected through multiple sectors, levels, and systems that together create an environment that influences household and individual decision-making, provision and access to services, and resources (1).

Reducing malnutrition in all forms requires promoting environments that enable “political and policy processes which build and sustain momentum for the effective implementation of actions that prevent or reduce malnutrition” (2). Enabling environments have “(a) knowledge, data, and evidence and its effective framing and communication; (b) political commitment, effective governance, and sound policy; and (c) leadership, capacity, and financing” (1).

Regarding nutrition knowledge, data, and evidence, what is most needed is that knowledge is available, accessible, and used regarding how to catalyze and implement what works at scale in a sustainable way in countries. Particularly useful are knowledge on nutrition measures; use of nutrition data; agricultural and other nutrition-sensitive actions’ progress, transparency, accountability, scalability, and sustainability; healthy and sustainable diets and food systems; and trade-offs (1). Access to credible and relevant research, and to local or tacit knowledge, is among the most important determinants of the use of evidence by decision makers (3). Making knowledge readily available increases access and use of evidence (4). Scientists and advocates of use of evidence who seek to make a difference to support evidence-based program and policy making will most likely succeed if they *1*) address how program managers, policy makers, and stakeholders think and consider how to respond, and *2*) consider the extent to which program managers, policy makers, and stakeholders control the programming and policy processes and have the power to influence those processes (5).

Models are needed for how to design and implement initiatives to make knowledge available through synthesis and generation; mobilize it through sharing, dissemination, and engagement with communities of stakeholders; and engender its use by program managers and policy officials to help countries make evidence-based decisions about nutrition and what works at scale in a sustainable way (6, 7). Interviews with 89 leaders or potential leaders in Kenya, Ethiopia, Bangladesh, and India found that leaders working in nutrition policy or programs had a range of technical knowledge about nutrition-specific and nutrition-sensitive interventions, but leaders not working directly in nutrition, particularly those in agriculture, had limited such knowledge (8). Leaders noted that knowledge was needed regarding multisectoral coordination, use of locally relevant and timely data, and implementation challenges. Leaders who were most capable were active and strategic in creating, commissioning, or translating new knowledge into examples or messages that would be locally understood and relevant to influence political decision makers (8).

Although India had made important policy decisions related to nutrition, many of which were evidence-based, coverage and quality of interventions has varied substantially across the country and little knowledge has been available as to how best to implement and scale up essential nutrition interventions. Furthermore, updated data on the nutrition situation in India have been limited because of infrequently conducted nutritional surveys, and online and face-to-face venues for stakeholders to share knowledge about nutrition in India operated in isolation and lacked resources. POSHAN (Partnerships and Opportunities to Strengthen and Harmonize Actions for Nutrition in India) was designed and implemented starting in 2011 to respond to the needs in India of program managers and national and state policy officials for knowledge about maternal and child nutrition. POSHAN was funded by the Bill & Melinda Gates Foundation to provide such information by making it available, mobilizing it, and engendering its use to facilitate evidence-based decisions. A political transition with the installation of a new prime minister occurred in 2014, partway through the POSHAN program period.

Given the need to understand how best to design and implement initiatives to help countries make evidence-based decisions about nutrition, this case study of the first phase of POSHAN in India aimed to investigate how availability, mobilization, and engendering use of nutrition knowledge in India was advanced to support evidence-based nutrition policy and programming from 2011to 2015 during a political transition.

## Methods

POSHAN in India was selected for this case study because it provided an opportunity to derive lessons from which other scientists and data-use advocates can learn. Use of nutrition knowledge for programming and policy making occurred in India through multiple processes, and POSHAN, in collaboration with knowledge networks and key stakeholders, contributed to these processes (9).

### Description of POSHAN

POSHAN was initiated to bring current evidence on maternal and child nutrition to decision makers and stakeholders and to facilitate decision making to support actions to improve maternal and child nutrition in India. The strategies and program components of POSHAN were designed during a 1-y inception phase in 2012, intended to understand how POSHAN could best support and strengthen use of knowledge of nutrition in India. Inception activities included mapping of state- and national-level stakeholders in nutrition in India (10) and reviews of existing knowledge systems and knowledge networks in India for their reach, use, and roles (11). The POSHAN model consisted of a strategy to improve the availability of, access to, and use of knowledge generated through a core set of research endeavors under POSHAN's key thematic content areas for knowledge generation identified during the inception phase, while also facilitating access to knowledge generated outside of POSHAN's activities. Together with partners, POSHAN produced knowledge and organized processes that yielded wide use of evidence. Subsequently, POSHAN strategically engaged and worked with partners, especially those identified as credible and influential through the inception phase activities. This engagement potentiated the use of POSHAN-generated evidence as well as broader evidence related to nutrition.

A key strategy was to link with influential knowledge networks to support demand-side activities, such as convening online and physical dialogues about evidence needs and solutions, and to enhance the diffusion of knowledge into practice. As one of many actors in the nutrition space, POSHAN sought synergies by catalyzing access to and uptake of nutrition-relevant knowledge that was beyond POSHAN's research portfolio (12). To synthesize existing knowledge, POSHAN conducted program and policy reviews about maternal and child nutrition at the national level and in selected states, compiled and featured findings from the latest global and national research studies through multiple platforms, supported the public launch of landmark global and national nutrition studies such as *The Lancet* Series on Maternal and Child Nutrition, developed primers on nutrition topics for nutrition stakeholders, and developed district data profiles on nutrition. To generate knowledge, POSHAN conducted primary research on the key thematic content areas identified as being most relevant to ensuring effective maternal and child nutrition actions in India; these included convergence between nutrition and health to deliver nutrition, working across sectors for planning and action for nutrition, implementing nutrition-specific interventions at scale, and strengthening the generation and use of data for tracking nutritional status and program operations. To mobilize knowledge and engender its use, POSHAN brought together diverse stakeholders who work at multiple levels and facilitated dialogues, learnings, and consensus building to advance the nutrition agenda in India; strengthened and linked existing nutrition networks and online portals and systems to promote knowledge sharing in nutrition; adapted to changing and emerging knowledge needs; and engaged with the media regarding nutrition-related issues and new findings.

### Political transition during the implementation of POSHAN

During the implementation of POSHAN, the policy and program environment nationwide did not remain stable economically and politically because of a major national political transition from September 2013 to May 2015 (9). The leading bureaucrats for the national nutrition program were substituted, and the opportunities of POSHAN partners to influence the government changed from those before the transition, bringing additional challenges for POSHAN. The transition had some consequences for the nutrition policy network such as slowdown or cessation of implementation, dysfunctional collaboration, loss of momentum, and uncertainty. These consequences occurred because of changes in personnel, priority changing away from nutrition, funds reduced, dynamics against predecessors’ work, community of stakeholders being fractioned, stakeholders’ inaction against weakened nutrition programs, ideologically based selection of nutrition allies, leadership and capacity building lacking among uninformed new governmental officials, change in the framework for decision making, and new ideologies with positions around nutrition issues not clearly defined (9). POSHAN continued planned national-level actions in collaboration with engaged stakeholders and state-level actions through partners and through direct involvement of the POSHAN team.

At the end of its first phase, in 2016, POSHAN received renewed financial support from the Bill & Melinda Gates Foundation for 4 y. The second phase continued to support policy and program decisions and actions to accelerate reductions in maternal and child undernutrition in India through an inclusive process of evidence synthesis, knowledge generation, and knowledge mobilization with emphasis on strengthening and amplifying POSHAN's state focus through most activities while also continuing to inform and engage with the national discourse on nutrition (13). This study reports only on the first phase, 2011–2015.

### Data collection

Data collection occurred during April and May of 2016. We selected a purposeful sample of national and state members of knowledge networks in India who identified themselves as users of nutrition knowledge and as part of program and policy communities. Interviewees were considered to be members of nutrition knowledge networks if they had ≥1 of several roles: strategic partner, government or other program manager, researcher or other knowledge generator, knowledge mobilizer (e.g., knowledge management officer), policy or practice community member, donor, ally, opponent, or advocate. We compiled secondary data in which we could verify the occurrence of events in India, the engagements among policy and programming communities, the enabled environment, and nutrition knowledge being available, accessible, used, and put into practice by knowledge networks. We conducted 37 semistructured interviews and assessed 1091 news articles, 318 Twitter and 175 Facebook posts, 12 YouTube events, 65 knowledge products, and 130 engaging events. The sample of interviewees were from government (*n* = 2, 5.4%), networks (*n* = 2, 5.4%), nongovernment organizations (*n* = 3, 8.1%), international development organizations (*n* = 6, 16.2%), private sector (*n* = 3, 8.1%), media (*n* = 2, 5.4%), civil society organizations (*n* = 5, 14.8%), research or academia (*n* = 7, 18.9%), and the POSHAN team (*n* = 7, 18.9%). The sample included 5 state-level interviewees.

### Interviews

The national and state stakeholders recruited purposefully participated in semistructured interviews. The POSHAN team members supported the interviews through an initial outreach to interviewees via e-mail informing them of the assessment being conducted by the authors, who had not been involved with the POSHAN initiative, and invited them to voluntarily inform the study during a face-to-face interview. E-mails were followed up by the second author until the interview was possible. The initial interview guides asked about roles in the India nutrition program and policy networks; sources and use of nutrition knowledge; experiences, opinions, and examples about knowledge being available, accessible, used, and put into practice; and how an enabled environment provided momentum for advancing evidence-based nutrition programming and policy making. The interview guides were updated during fieldwork, and a guide for key informants was added. The updating process continued with the validation of interview guides with 2 key informants. Interviews were audio-recorded, and quotes were transcribed.

The University of South Carolina Institutional Review Board determined that the study was exempt from the Protection of Human Subjects Regulations. Interviewees consented to be interviewed; participation was not a condition of employment. Interviewees were informed that their reports were confidential and that recordings were only accessible to the authors. Upon completion, interviews were assigned a consecutive number to protect the identity of interviewees and affiliated institutions; the second author was responsible for data security. The names and the institutions of interviewees are not disclosed in any way; interviewees are only described by the categories of the sectors they represent.

### Documents and events

We obtained POSHAN documents including POSHAN's strategy, progress reports, and the complete set of POSHAN knowledge products. Information on POSHAN events was organized in chronological order.

### Media

We sampled traditional media news included in the POSHAN's news database, news articles produced by the participants of the POSHAN fellowship for journalists, and additional media news articles searched for assessment purposes by using predefined term-categories. The search term-categories used included POSHAN's 4 thematic areas, the state partners’ proper names, the states POSHAN targeted including New Delhi, and the proper names of the POSHAN team members whom interviewees mentioned at least once. This additional searching process was completed to assess what other news could complement the news dataset for triangulation purposes. This search was completed using the Indian edition of Google news in English, restricting the search for events that occurred between 2011 and 2015.

English content was assessed from 3 social media platforms from 2013 to 2015: POSHAN's Twitter page, Facebook timeline, and International Food Policy Research Institute (IFPRI)-POSHAN's YouTube channel. The POSHAN social media platforms were launched in 2013. A health communications expert supported the definition of methods for using media data and the data collection for traditional media, YouTube, and social media (Facebook and Twitter). Sampled media were only used for the triangulation and process tracing methods in the analysis related to the multisectoral strategy.

We accessed the POSHAN website (http://poshan.ifpri.info) to confirm access to all the knowledge products. The website showed a link to past and upcoming events and topics. It had a search engine for finding products, events, and other information by entering keywords. The website showed contact information including a physical address, phone and fax numbers, and an e-mail address; had a Twitter feed; and allowed connecting by subscribing to the mailing list, and by joining the conversation on Facebook and through Twitter. Website visitors could connect by using the “FeedBurner” tool for receiving content updates in My Yahoo!, Newsgator, Bloglines, and other news readers.

### Data analysis

Data were analyzed using open-axial coding, and content and themes analysis. To examine how nutrition knowledge was made available, mobilized, and engendered for use and how it enabled a changing political environment to support evidence-based nutrition policy and programs, all primary data as transcripts from interviews were organized in an Excel file and coded according to criteria to assess whether the processes of evidence-based programming and policy making and enabling environment had occurred and how these processes happened (2, 5, 9). Interview data were analyzed in 3 rounds using open coding to identify emerging themes, categories, and subcategories (9, 14, 15). During the first round of analysis, interview data were coded for the themes addressing the study aim in 2 steps: first, how availability, mobilization, and use of nutrition knowledge was advanced to support evidence-based nutrition policy and programming, and second, how use of nutrition knowledge advanced an enabling environment to support evidence-based nutrition policy and programming during the prime ministerial transition. During the second round of analysis, data were coded for 2 subcategories: evidence-based policy and programming addressing how stakeholders think, consider how to respond, and account for influence and power; and how those stakeholders adopted practice changes when further facilitation and a follow-up occurred. A last round of data analysis was completed to inform emerging themes on perspectives on, trust in, and coverage of the knowledge initiative. Data were coded under 4 emerging subcategories of critiques. Content and themes analysis was used for POSHAN documents, media, and events for triangulation and process tracing. Processes were initially drafted based on the POSHAN's paths for evidence-based programming and policy making and enabling environment (12). Processes were updated and finalized according to reports from study participants and the emerging themes in secondary data sources to maximize describing the actual processes as these occurred (9). We present primary data as interview quotations.

### Triangulation and process tracing

A subsample of all data sources providing information on the evidence-based India multisectoral strategy for nutrition of the Ministry of Women and Child Development was used for triangulation and process tracing methods (15, 16). The purpose of using a subsample for triangulation and process tracing was to confirm the processes of evidence-based programming and policy making that occurred drawing on multiple data sources (interviews and all secondary data) and to examine if evidence-based policy and programming (using the multisectoral strategy as an example) could be linked to POSHAN. The multisectoral strategy was selected for this analysis because it corresponded to a POSHAN thematic area and to a governmental-led effort. The multisectoral strategy was a key government milestone in India's multisectoral endeavors to improve nutrition (17). This government effort advanced multisectoral work on nutrition, meaning: “working more comprehensively to bring the nutrition policies, programs, resources and actions to bear at the same time and place on the same child.” Following a National Council recommendation, the multisectoral program in 200 high-burden districts was planned with sectoral actions for different ministries and setting up nutrition councils at state and district levels (17). The multisectoral strategy in India was launched in early 2014.

Triangulation was completed by using the subsample providing data on the multisectoral strategy: 22 semistructured interviews, 10 documents as knowledge products, 22 engaging events, 5 news articles, 19 Facebook and 37 Twitter posts, and 10 YouTube events. Analysis of themes was used for interviews, and content and themes analyses were used for all secondary data sources. The themes emerging from all data sources were compared against the criteria to determine if availability, mobilization, and engendering use of nutrition knowledge and enabling of the environment during the political transition occurred in relation to the multisectoral strategy and how it occurred (2, 5, 9, 17). Triangulation showed that, in relation to the multisectoral strategy, the use of evidence occurred as knowledge access, engagement of policy and program communities, informed dialogue, decision making, and strategic documents (9). Use of evidence for practice changes was limited due to events related to the prime ministerial transition and the subsequent changes within knowledge networks and to the policy and program communities that included the reassembled governmental technical teams. Process tracing was used as a within-case method of analysis (9, 16, 18, 19) following the chronological sequence of nutrition-related events and POSHAN's events around the multisectoral strategy to determine if these could be linked. A narrative of ordered occurrence of events related to POSHAN was developed. An event was defined as an occurrence having significance for the development of the multisectoral strategy. Process tracing by using the chronological sequence of nutrition-related events was linked to POSHAN's implementation and specific events, products, and milestones (9). Triangulating of stakeholders’ reports during interviews with all other data sources, and process tracing maximized trustworthiness.

## Results

### Analyzing the landscape and making knowledge available

**FIGURE 1:**
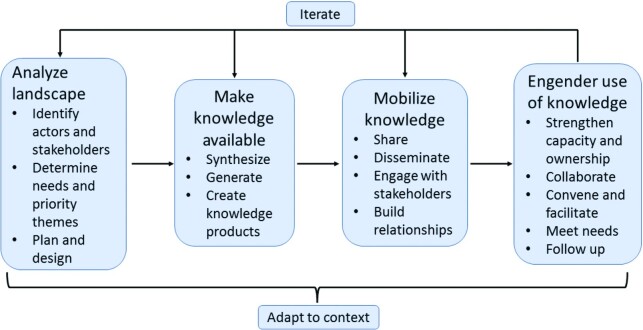
Advancing the use of nutrition knowledge to support evidence-based nutrition policy and programs through analyzing the landscape, making knowledge available, mobilizing it, and engendering its use.

Preceding POSHAN's design, initial inception activities assessed the landscape of nutrition stakeholders for addressing undernutrition in India, identified actors and their influence power at national and state levels, and developed strategic priorities for making knowledge available, mobilizing it, and engendering its use (**Figure 1**). Stakeholders were assessed for actor power, political context, ideas, and narratives (20). The landscape analysis addressed how to influence the framing of debates; get issues on the agenda to influence awareness, attitudes, or perceptions; affect language and rhetoric; open new spaces for dialogue; and promote consensus (21). POSHAN also identified in 2012 the 4 priority thematic areas in collaboration with knowledge networks and strategic partners. POSHAN made knowledge available (**Box 1**) by creating multiple forms of knowledge products to be disseminated on virtual platforms and during in-person events. Knowledge products included policy, program, and research notes; journal articles; update bulletins; presentations; blogs; and newsletters.

### Mobilizing and engendering use of nutrition knowledge by engaged policy and program communities

The processes to advance use of nutrition knowledge in India as facilitated by POSHAN from 2011 to 2015 to support evidence-based nutrition policy and programs went beyond generating evidence packed in ways that were easy to understand. The processes addressed the ways program and policy stakeholders think and demand evidence and responded to those demands by taking into consideration their power to influence processes (5). Specifically, the evidence was relevant to influential stakeholders who valued and trusted the science, understood the evidence, and had the motives and the opportunities to act on it (Box 1). POSHAN helped bring stakeholders together to facilitate knowledge access and engagement for effective knowledge sharing, reach consensus, and shape evidence-based framing of nutrition issues and solutions. These processes helped to improve nutrition as led by the individuals and the institutions represented among the engaged program and policy communities.

Mobilizing and engendering use of nutrition knowledge by engaged policy and program communities were related to a sequence of nutrition-related events. Key reported features about the process facilitated by POSHAN were meeting evidence demands, including thematic areas that coincided with governmental efforts, and facilitating the timely use of knowledge, advancing consensus, knowledge flow reaching state levels, participative processes for informing the knowledge networks, and timely availability and access of key pieces of knowledge needed. Reports on ways the evidence was accessed included direct engagement events at state and national levels through online web, own initiatives (to access and interpret nutrition knowledge), e-mails, newsletters, and direct contact with the POSHAN team and as part of knowledge networks and key stakeholders.

Box 1 **Quotations regarding processes by the knowledge initiative to make knowledge available, mobilize it, and engender its use through facilitation and follow-up**

“POSHAN occurred in 2011–2014; they had something [a product] on multisectoral [work], with strong advocacy and rooted to reality. IFPRI and others contributed for the changes needed, providing assistance to the planning commission.” (I-29)

“The way we worked with POSHAN, finding venues to informing the debate, because we care about informing the dialogue with evidence, bringing evidence to policy makers, using opportunities to feed in policy and program decisions, e.g., based off the UP-landscape analysis of nutrition stakeholders; when their nutrition mission took off, they used the information we had to inform their nutrition mission.” (I-36)

“[Regarding] the working groups on essential nutrition interventions for designing recommendations, POSHAN brought in intellectual processes, and the involvement of experts for a common goal. POSHAN and others have a mandate on science-based evidence that has contributed into the network's strengthening, e.g., State Nutrition Missions. They took it to the next level at influencing the agenda by bringing people together and [potentiating] evidence-based decision making. POSHAN has created links to different partners for advocacy and has leveraged groups.” (I-10)

“Block-level people need training and to be walked through the data, that role should be taken by the system within government but not possible, that is where NGOs and civil society organizations are there to help, not enough though.” (I-14)

“POSHAN has produced good quality work and has contributed to bringing stakeholders together; but leadership, capacity building, and within governmental systems changes need to be potentiated.” (I-5)

“With policy makers you have to take the time to walk them through the data. POSHAN has attempted to educate policy makers through workshops, interactive meetings, and education sessions. Only sending them documents does not help, you need interacting meetings.” (I-24)

The mobilization of knowledge accelerated by the end of 2013. The use of knowledge among engaged stakeholders required facilitation and follow-up to maximize knowledge understanding, contextualization, and application. Use of knowledge occurred in 2 ways: as knowledge use for the creation or updating of evidence-based strategic documents and as knowledge use for practice changes. Knowledge use for practice changes occurred in specific instances only after the use of knowledge informed dialogue, decision making, and the creation of strategic documents along with leadership, will, shared commitment, and movement of discourse to action. Knowledge use within the government was engendered by presenting district nutrition profiles for strengthening breastfeeding promotion activities and for informing frontline worker planning and practices, and governmental testing for the multisectoral strategy before the government launched it at the national level. Other processes of using knowledge to change practices included those facilitated within specific sectors (such as networks and civil society) described as a timely use of evidence for informing key policy and programming efforts appointed by the government and creating more simplified and usable materials for action reaching a larger audience.

### How use of nutrition knowledge supported evidence-based nutrition policy and programs during political transition in 2014

The policy and program environment nationwide became economically and politically unstable because of the major national political transition. Ten years of relative political stability characterized by important nutrition policy making and the adoption of guidelines or plans—infant and young child feeding and control of anemia (2004), Food and Nutrition Security Commission (2006), vitamin A and zinc (2007), and management of children with severe acute malnutrition (2011) (17)—was followed by a burst of change in the landscape for addressing undernutrition in India that occurred around the time of the national elections and the transition to a new prime minister.

Events related to national elections and the takeover of the new prime minister occurred in 2014, including changes in the national policy landscape of actors for nutrition, ministerial and bureaucratic changes, and transitions in the nutrition coalition (**Box 2**). This transition prompted some stakeholders to discontinue their contribution to the mobilization and use of knowledge, other stakeholders joined the technical teams (new stakeholders), and others continued either in the same or a different institution. The influential power of key stakeholders changed. For example, grassroots groups and civil society were reported to be less influential than they were under the previous prime ministerial administration.

Box 2 **Quotations regarding challenges to support evidence-based nutrition policy and programs during political transition in 2014 and the role of the knowledge initiative**

“Even though the landscape of nutrition is the same [at the time of data collection], and the departments in charge of nutrition are the same, the decision-making landscape has significantly changed; politically the decision-making framework now is completely different in terms of what and where you need evidence, who is the person in the government through whom processes initiate, and the person who makes the final decision. They [organization] has stopped doing things [sic], need to recalibrate the message, to reframe things, and in some cases, they had to start all over again.” (I-19)

“The current Prime Minister and government are working on a Nutrition Mission, and there is a Nutrition Strategy coming out soon. They are committed, but the gaps on implementation are the issues now. How to get the government to work and deliver for a permanent solution? They are looking at why things have not worked. I would have liked them to act faster, they did not automatically follow what the previous government was doing” (I-28)

“There is an issue on information. There is [someone] that disputes the figures of malnutrition in India … does not know this [nutrition] is an issue … is not linking water, sanitation, and hygiene (WASH) to nutrition.” (I-22)

“The new political leadership, a mind change on nutrition priority, and budget allocation. Should we focus now on the Ministry of Health? Should we create an independent nutrition [office]? Should the private sector fill in gaps? There is a transition towards corporate responsibility; is nutrition an interest to them? [There are] new views in terms of convergence, migration, gender, data for decisions, and on what is cost-effective.” (I-16)

“POSHAN was involved in the process of starting all over again because the new minister requested a nutrition strategy. [The minister] appointed out a committee, they accepted our inputs, they completed a nutrition strategy but then there were politics at the ministry. We were part of the minister's technical strategy group. We co-wrote a strategy document for nutrition with the targets, and with nutrition-specific interventions; but we do not know what the final document will look like.” (I-32)

“POSHAN is seen as neutral [by the government and others] we are not neutral. Having a neutral POSHAN as an advocate for communicating with the government they can play a role that is technical-and-nobody's side but [a side] that is good for nutrition. If POSHAN creates a space, then we can use it; it could be, the government seems more open to listen to experts than to grassroots. But it seems like POSHAN has not succeeded either.” (I-14)

“IFPRI-POSHAN … does not exist to inform one person, [not even] the Prime Minister. It exists to cultivate a platform for the long term, to informing the people that can influence whoever government is in power. The scientific community can influence that way: bringing people together, bring in data. There was a huge vacuum nobody was filling, somebody should fill it, and POSHAN has. As a research institute they have to keep the distance and keep it objective [The opportunity is to] engage with new stakeholders, major institutions for [continuing] the dialogue, so in the long-term policies keep in place. Regardless of the changes in politics and in the discourse, it is a matter of keeping the powerful people nutrition-literate. Inform nutrition-science and not nutrition-ideology. Inform on what is relevant and produce knowledge to act upon.” (I-12)

“When information was needed if not immediately available, there was something in the back among the vast [IFPRI-POSHAN] resources that has been useful and available for discussions with government in a very short span of time.” (I-3)

In this context, the POSHAN team, knowledge networks, and stakeholders potentiated the flow of nutrition knowledge to some reassembled technical teams and to an influential group of stakeholders that remained constant from 2012 to 2015 and contributed in particular ways to benefit the processes advancing use of knowledge to inform policies and programs among the new administration and collaborators (Box 2). The transitional events created short-term needs for nutrition evidence within the new administration that POSHAN and stakeholders were ready to meet and performed effectively toward generating and mobilizing the nutrition knowledge in response to demand to inform programs, policies, and practices. After the political transition, reported processes facilitated by POSHAN included making knowledge ready for the reassembled nutrition technical community to engage, and contributing in a timely manner to meeting specific information requests from the new government. Key knowledge needs that were met with short notice were reported to have advanced the institutionalization of the Coalition for Sustainable Nutrition Security in India, the creation of a multisectoral strategy, cost-effectiveness analyses, the National Nutrition Mission Strategy, and state-level response to mandatory nutrition missions. Use of knowledge to inform nutrition policy was advanced by POSHAN's facilitation of key opportunities to have an influence after the transition because of IFPRI's standing as a research institute, the perception of being neutral, the ongoing actions at the state level, and readiness to provide information to the reassembled technical teams appointed by the new government. Some study participants suggested caution to claim any early successes on the continuity of evidence-based programming and policy making right after the political transition.

### Perspectives on initiative to make knowledge available, mobilize it, and engender its use

The terms and expressions study participants used to refer to the POSHAN knowledge initiative included: nonconfrontational, genuinely interested in the issue, not seeking a protagonist role, leaders for advice about scientific evidence, nutrition champions, scientists doing quality work, evidence-informed, unafraid, unbiased, objective, with no agenda, standing by what the data show, locals or Indians, working according to opportunities, rigorous research organization, advocacy group, and influential with the Indian government (**Box 3**). POSHAN was viewed as providing relevant information that addressed gaps in India in terms of data overall, multisectoral work, essential nutrition interventions, cost-effectiveness, and district profiles. It was also reported as pointing out what to read. Characteristics about POSHAN making a difference that emerged from reports were credibility, objectivity, the perception that the team could be trusted nutrition partners and champions, the team's attitude, and what the team “intrinsically” represented.

Some critiques and disagreements in relation to the process of mobilizing and engendering use of knowledge as facilitated by POSHAN were reported by study participants (Box 3). They mentioned obstacles for improving evidence-based programming and policy making. Specifically, there were reports of the process not being trusted due to POSHAN's donor organization and particular sources of data used to inform policies and programs. Others reported remaining gaps not addressed by knowledge products such as operational evidence about how the implementation of proven nutrition interventions works and how to harmonize ministries and address operational challenges. There were instances of perceived exclusion from processes generating and mobilizing knowledge and engaging stakeholders, and of perceived biases in the process of generating and mobilizing evidence toward local activism and away from particular interventions of interest that are proven to be cost-effective and scalable interventions such as food fortification. There were reports of POSHAN not being indispensable, being one of many actors, and being not enough for nutrition landscape changes in India.

Box 3 **Quotations regarding perspectives on, trust in, and coverage of knowledge initiative**

“POSHAN has put high quality knowledge in one place. Before nobody was playing that role of bringing evidence and research on India in a digestible form. POSHAN is generating knowledge and bringing stakeholders together at the national level while keeping an independent role, away from bureaucrats, and directing the attention back to the evidence. It has created momentum at the national level. Different from others, I know when reading a POSHAN product it will never be suboptimal. I question the work by others: look at methods, sources, analyses; no need to do that with POSHAN. A body like that is needed. They are not confrontational and say what needs to be said. They do not shy away from it. They bring credibility to the discourse.” (I-9)

[Regarding evidence-based programming at the state level] “As the only nutrition specialist in my office, by referring to POSHAN products I was able to raise the profile of nutrition even within my own office-teams. The district level profile was the first of its kind, before that there were no district-level data. POSHAN is a brand-name; if they do it, people will trust and listen.” (I-24)

“I am not a big fan of POSHAN. Money from [POSHAN donor], there is a problem of conflict of interests. We have a policy that we do not take money from [that donor], so we have a problem using the products. I am critical about the use of the gaps and cost analysis products replicating the [country] analysis, using a model including mass media campaigns. I do not agree with generating data based on these experiences. It is not sustainable. Building the capacities of workers and systems, those models would be sustainable.” (I-20)

“You can't allow personal biases driving the agenda; it needs to be evidence based. The POSHAN evidence agenda ignores food fortification, one of the most cost-effective and scalable interventions; it is biased towards local activism.” (I-21)

“Regardless our strong voice on ready to use foods, we are excluded now from the conversation. It has to do with being associated with the previous government; [we] lost space we feel excluded from the expert-groups, unable to engage and minimized to be an implementer. When [POSHAN] state products are presented at high levels we feel pushed aside [not given credit]. We take the harder line IFPRI refuses to take. They go far but not further. The way to reach children's policy for IFPRI is research, [for us] is shifting power.” (I-8)

“What is needed is operational evidence. For example: the 10 recommendations for children are okay, these are not under question; that breastfeeding works is not the question but how is it that works in context, how to bring it up-scale this is the critical evidence needed, what it is of the government's interest. IFPRI has done it up to a certain level. We already have the critical policies in place, now we need to know how to implement them, what are the standards for operationalization procedures, test them, and document that, e.g., data on multi-sectoral work is good but what is next, how to do it while implementing programs, this is the new evidence to generate.” (I-10)

## Discussion

The POSHAN knowledge initiative contributed to evidence-based nutrition programming and policy making and helped enable the policy environment in India by making knowledge available, mobilizing it, and engendering its use through engagement of policy and program communities, informed dialogue, consensus, and decision making. POSHAN effectively identified key stakeholders in nutrition and knowledge networks and provided nutrition information to support decisions by synthesizing, generating, and mobilizing nutrition knowledge. POSHAN contributed to the engagement of policy and program communities with nutrition issues and with processes of knowledge generation and to the creation of an enabling environment for effective knowledge sharing.

The POSHAN model advanced traditional knowledge management principles to not only make knowledge accessible as needs emerged and to reorganize, reformat, and translate knowledge into usable products, but also to convey clear messages addressing how stakeholders think, bringing in considerations on how to respond, and strategically addressing the influential power of the audiences (5). POSHAN made accessible relevant, good-quality research in a timely manner and facilitated engagement and collaboration for the policy and practice communities to use knowledge for decision making, in alignment with previous studies on use of evidence by policy makers (3, 22). This experience addressed a pivotal aspect of the policy process in which scientists inform rigorously the identification of issues as much as that of solutions through a solid collaboration with program managers and policy makers who comprised the knowledge networks and the key stakeholders jointly navigating the politics and contingencies of program implementation and policy making (7).

During the implementation of POSHAN, a major national political transition occurred in India. Political transitions often make sustaining initiatives, policies, and programs difficult because of shifts in political priorities, interruption of government operations, changing actors, and collaborations becoming dysfunctional (23). The institutional and political environments during the transition in India with subsequent changes within knowledge networks brought about challenges but also created opportunities. POSHAN in collaboration with knowledge networks and key stakeholders was able to establish and restore relationships with the new set of actors after the transition and to meet immediate needs of nutrition information for making decisions early during the first term of the new prime minister. This Indian experience of scientists and policy and program communities facing bursts of instability and change while seeking continuity of use of knowledge aligns with the punctuated equilibrium theory (24) cited by experts highlighting the necessity to know well the policy process if scientists and data use advocates seek to effectively support evidence-based programming and policy making (5). The punctuated equilibrium theory helps to understand the process of change in a complex social system. Changes often include sudden shifts in how policy makers and other influential groups understand a problem, to what evidence they choose to pay attention, and how they use that evidence (5). Often a complex policy process becomes even messier and more unpredictable than it was before the burst of instability resulting from a political transition. Adapting and effectively responding to the short-term emerging windows of opportunities to maximize continuity is possible, as documented with this case of POSHAN in India and in other countries (9, 23).

Study participants provided perspectives about the POSHAN knowledge initiative and the processes for making knowledge available and mobilizing it, which created both opportunities and obstacles for evidence-based programming and policy making. These perspectives highlight a lesson for those navigating messy and unpredictable policy processes and making choices about pragmatic ways to adapt and engage by seeking allies and creating coalitions (5, 25). In this case POSHAN engaged with networks of policy and program communities and key stakeholders. Naturally, those who join any process and choose to actively engage and participate likely share a fraction of the dominant ethical values, beliefs, and perspectives with other participants and with facilitators for understanding issues and solutions. As reported by participants, data and scientific evidence informed technical conversations, but factors intrinsically linked to POSHAN (e.g., its donor) prevented some key actors from engaging with or joining the conversations.

A lesson from this experience is the importance of incorporating in an inception phase some self-assessments to understand how scientists and data advocates are perceived and assessments of unconscious bias to allow a robust component of reflexivity (15). Reflexivity is a qualitative method to document self-awareness including political, cultural, ideological, and social consciousness toward self-questioning and self-understanding about one's own perspective and voice (15). Adopting such assessments early in the process will bring about improvements to participative processes of knowledge generation and mobilization in ways that maximize uptake by both allies and opponents. This experience in India also suggests that some key actors will be reluctant to participate, hence facilitators may need to consider innovative ways to have them join the conversation and to avoid missing out contributions that will potentially bring about breakthrough improvements for success.

The POSHAN knowledge initiative suggests a planning and implementation model for other countries regarding the essential steps to undertake to successfully support decisions through synthesis, generation, and mobilization of nutrition knowledge. POSHAN specified a strategy, work plan, objectives, and activities. POSHAN was adaptable, responding in a short time to a new landscape of nutrition stakeholders and priorities. The inception phase was used to obtain the information required to make these initial specifications. POSHAN advanced targeted and tailored knowledge translation (6) to best fit the characteristics of users by first identifying the kinds of nutrition knowledge needed by specific program and policy communities in India and then moving on with knowledge synthesis, generation, and mobilization. These steps are replicable in other countries. Although the activities undertaken will likely differ somewhat across countries, the steps that lead to the specification of activities likely apply universally and would serve to maximize success.

Besides having a useful planning and implementation model, POSHAN exhibited several elements important to its success. POSHAN had creative and effective leadership, staff, and funding to carry out its work; expertise to manage the nutrition content; skill and experience needed to navigate the social and political context in which the initiative was working and to build positive relationships; willingness and skill to use information technology; and a variety of venues and media, and institutional support by a widely recognized organization in India (i.e., the IFPRI). POSHAN adapted and was highly responsive to emerging knowledge needs during and after the political transition and built momentum for nutrition knowledge that reflected global and in-country nutrition priorities. POSHAN functioned as a trusted, nongovernmental entity that created demand for knowledge and met that demand through responsive knowledge synthesis, generation, and mobilization. The POSHAN team was perceived as trusted, neutral, genuinely caring for addressing nutrition issues in India, local (i.e., Indians who understood Indian context, culture, and history), and performing high-quality work meeting both international and local standards for a research institute. Even those reporting concerns about the influence in India of POSHAN's donor did not question the initiative's work in effectively providing nutrition information to support decisions. Replicating all these elements in other countries would be challenging but not impossible. The POSHAN experience supports evidence that investments toward translating knowledge into usable products and being intentional in the engagement of knowledge users (26, 27) pay off, organizational culture (28) matters for success, and using windows of opportunity (5, 22) maximizes effectiveness.

Prior guidance for academics seeking to influence policy making has highlighted the importance of high-quality, relevant, and readable research that is accessible to policy makers; that those producing the knowledge understand the policy process, decide to adopt a role of advocate or honest broker, and engage routinely and develop relationships with knowledge users; and continuous reflection regarding how the process is working (26). From experience incorporating farmers’ voices into policy development has come guidance about how to effectively communicate to make research relevant to policy makers, develop and maintain relationships with them, use windows of opportunity, and adapt presentation and communication for the audience (22). A model for bringing evidence to policy making called Policy Lab was developed that acknowledges the importance of evidence per se while emphasizing that the producers of the evidence and its users collaborate and navigate through experiences, values, and perspectives (27). This Policy Lab model involves establishing venues for open and honest conversations on a policy topic; creating networks, collaborations, and partnerships; and making the evidence available in a user-appropriate format that is timely (27). The model used by POSHAN in India is consistent with this guidance and experience from the Policy Lab, providing an alternative model to the latter. Furthermore, the POSHAN knowledge initiative advances our understanding on how knowledge producers can adapt to and be effective in a changing policy and political landscape for the continued use of knowledge as the knowledge needs of nutrition stakeholders evolve.

POSHAN incorporated systematic and robust internal documentation of planning and implementation of its model, which aided the authors by complementing the use of existing data with primary data to investigate how and how well POSHAN had worked (9). This practice could be adopted by others and help minimize challenges faced by knowledge management initiatives in obtaining evidence of success and learnings that are important for bringing together evidence and policy making (27) and justifying that nutrition knowledge management initiatives are investments with returns worth considering for donors seeking to maximize evidence-based nutrition policies and programs. Substantial data of multiple types were collected allowing for integration and triangulation that strengthened trustworthiness. Although the single-case design used cannot provide a formal counterfactual to definitively establish what would have occurred if POSHAN did not exist, we used process tracing (9, 16, 18, 19) and direct inquiry to informants to establish the contributions that POSHAN made.

Low- and middle-income countries like India will be increasingly faced with simultaneously responding to the need to improve population health by reducing undernutrition and preventing chronic disease resulting from overweight, obesity, and unhealthy dietary intake. Making nutrition knowledge available, mobilizing it, and engendering its use can be advanced through knowledge initiatives to support evidence-based decision making in a large and complex country even during a political transition.

## Notes

This study was funded by the Bill & Melinda Gates Foundation, through Partnerships and Opportunities to Strengthen and Harmonize Actions for Nutrition in India (POSHAN), managed by the International Food Policy Research Institute. This study was internally commissioned.

Author disclosures: The authors report no conflicts of interest.

The POSHAN team in India requested the study, helped conceive and design it, and assisted with initial contacts to interviewees but played no other role in data collection or analysis. Decisions about results, interpretation, and conclusions rested with EAF and JLE-A, who designed and conducted the study.
